# The Impact of an Undergraduate Community‐Based Medical Education Program in a Japanese Urban City

**DOI:** 10.7759/cureus.54204

**Published:** 2024-02-14

**Authors:** Satoshi Hiramine, Masaharu Nagata, Mosaburo Kainuma

**Affiliations:** 1 Community Medicine Education Unit, Graduate School of Medical Sciences, Kyushu University, Fukuoka, JPN; 2 Internal Medicine, Shin-eikai Hospital, Kitakyushu, JPN; 3 Japanese Oriental Medicine, Graduate School of Medicine and Pharmaceutical Sciences, University of Toyama, Toyama, JPN

**Keywords:** educational settings, an urban city, community healthcare, community based medical education, medical students

## Abstract

Introduction: With an aging global population posing healthcare challenges, especially in community healthcare, training professionals for community healthcare remains a global educational challenge, including in Japan. While previous research shows increased student interest in community healthcare through practical experiences, the focus has primarily been on rural areas, leaving a gap in understanding urban-based community medical education. This study aimed to evaluate the impact of urban community-based medical education on students' understanding and attitudes toward community healthcare.

Methods: This study was conducted at Kyushu University in Fukuoka City, the sixth-largest city in Japan. For fifth-grade medical students, a community-based medical education program is mandatory, with a one-week schedule that includes orientation, one day in a clinic, and three days in a community hospital. The program aimed to expose students to various healthcare settings, emphasizing outpatient care, inpatient management, home health care, home nursing, and rehabilitation. A self-administered questionnaire referencing the Model Core Curriculum for medical education was administered immediately before and after the program, and the differences in students' perceptions were assessed using the Student's t-test.

Results: Findings from 188 students completing both pre- and post-program questionnaires revealed significant improvements in perceptions of students' knowledge and skills related to community healthcare. After the training, there was a noteworthy positive shift in attitudes toward community healthcare and increased interest in primary care physicians as a career choice. Although students maintained a preference for urban areas, there was a notable inclination towards rural settings. The study highlights the program's success in enhancing students' understanding and positive attitudes toward community healthcare within an urban context. It challenges prior notions regarding work-life balance and urban-rural preferences in career choices.

Conclusions: Urban community-based medical education significantly improved students' perceptions and attitudes toward community healthcare. It offers valuable insights for curriculum planners, emphasizing the need for continued research into sustained effects and broader applicability.

## Introduction

Community healthcare is an essential societal system worldwide, facilitating healthy and secure lives for people [1]. It functions as a healthcare system addressing major local health concerns, offering comprehensive health promotion, prevention, treatment, and rehabilitation services. As aging societies have been progressing globally, including in Japan, community healthcare has become more intricate due to the increased number of patients with multiple chronic conditions and polypharmacy, emphasizing the growing significance of primary care [2,3].

Training healthcare professionals who support community healthcare remains a central challenge in medical education worldwide, including in Japan [4-6]. Previous research indicates that the number of students considering a career in community healthcare increases when they gain more practical experience in community healthcare settings during community medical training [7-9].

In Japan, as well to train primary care physicians, community-based medical training experiences, akin to global trends, are incorporated into the curriculum of nearly all medical schools. Additionally, the Model Core Curriculum for medical schools strongly advocates for community-based medical education [10].

Reports suggest that students, through clinical training in communities, experience patient-centered healthcare, interprofessional collaboration, and community-oriented primary care, heightening their inclination towards community healthcare [11,12]. However, existing studies predominantly focus on clinical training in depopulated areas, rural regions, and remote islands, with limited documentation on the effectiveness of community-based medical training in urban areas.

Hence, the objective of this current research is to investigate the effectiveness of community-based medical education conducted in urban areas by analyzing the questionnaires given to the students immediately before and after the practical training. The impact of medical education on students can be influenced by the learning environment they experience [13]. Moreover, regions encompass not only depopulated areas but also extend to large cities, where comprehensive care becomes a significant concern due to aging populations [14]. This study aims to ascertain whether there are tangible training effects when community-based medical education is conducted in major urban areas, shedding light on its efficacy for fostering the personnel essential to support community healthcare care. Consequently, these findings are anticipated to serve as crucial insights for curriculum planners contemplating the location of community-based medical education.

## Materials and methods

Setting

Fukuoka City, where Kyushu University is located, is the sixth-largest city in Japan. Since the first national census in 1920, the population has consistently increased for males and females, reaching approximately 1.6 million in 2020. Japan has one of the most aged populations in the world, with 28.7% of the population aged 65 and over in 2020. The aging rate in Fukuoka City is also continuing to increase and has reached 22.1% [15].

The Community Medicine Education Unit, Graduate School of Medical Sciences, Kyushu University, was established in 2012 and started organizing community-based medical education programs. For fifth-grade medical students, practical training in clinics and community hospitals is mandatory. The goals of this program encompass gaining expertise in symptomatic diagnostics and community healthcare, acquiring knowledge about conditions and diseases prevalent in the elderly population, understanding the dynamics of interprofessional collaboration, and fostering the motivation to assume responsibility for community healthcare. To achieve these goals, we requested the cooperation of clinics that provide outpatient and/or home health care as primary care physicians and community hospitals with both acute care and convalescent and/or chronic care wards. In 2018 and 2019, 13 clinics and 13 hospitals participated in this program: 12 clinics and six hospitals are located in Fukuoka City, with the remainder all located in the suburbs of the city.

**Figure 1:**
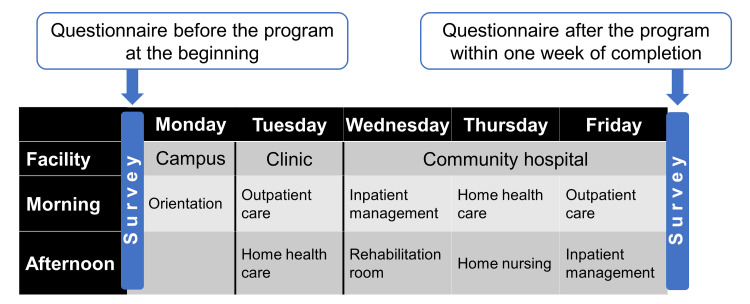
Time schedule and an example of the learning contents in this program

Figure 1 illustrates the time schedule and an example of the learning contents of this program. The program was scheduled for one week, with staff of the Community Medicine Education Unit explaining the goals of the program to the students on the first day, after which the students spent one day in the clinic and three days in the hospital. The program includes a variety of clinical placements in outpatient care, home health care, home nursing, and rehabilitation. We decided to allocate more days for training at the hospitals compared to the clinics because of the learning contents, such as the emergency department, inpatient management, and pre-discharge team conferences, which were not readily accessible in the clinic setting. While certain students may solely observe healthcare professions, the majority will actively participate in these domains. For example, they take patient histories, perform physical examinations, and execute medical procedures such as blood pressure measurements and intravenous blood draws. In order to increase the probability for each student to experience these learning contents in practice, and with the intention of fostering a sense of responsibility and professionalism, students were placed alone, not in groups, at each medical facility. In order to ensure uniformity of training content among facilities, an annual faculty development meeting is held by the Community Medicine Education Unit.

Participants

This study was conducted from April 2018 to March 2020, and all fifth-grade medical students were included. They had completed the lectures on the fundamentals of clinical medicine and had successfully passed computer-based testing and an objective structured clinical examination during their fourth year. The exclusion criteria for this study were the presence of deficiencies in one or both of the two questionnaires.

Questionnaires

For this study, a self-administered questionnaire was developed referring to the Model Core Curriculum for medical education in Japan [10] and previous studies [16,17]. It consisted of 13 items assessing perceptions of medical knowledge and skills that were thought to be fostered by community medicine practice and six items assessing attitudes toward community healthcare (Table 1). All items were rated on a scale of 0 to 100, where "0" indicated "unconfident" and "100" indicated "confident" for understanding medical knowledge and skills (Items 1-13). Similarly, for attitudes toward community healthcare (Items 14-18), "0" represented "disagree," and "100" represented "agree," except for Item 19, where "0" denoted rural areas and "100" denoted urban areas. The questionnaire was made available on the faculty's digital platform and administered to students at the beginning of the orientation and within one week of the completion of this program (Figure 1).

**Table 1:** Survey questions before and after the program All the answers were on scales from 0 to 100. For students' understanding of medical knowledge and skills, "0" was for "unconfident" and "100" was for "confident". For students' attitudes toward community healthcare, "0" was for "disagree" and "100" was for "agree", except for Item 19 (*), with "0" being rural areas and "100" being urban areas.

Items to assess students' understanding of medical knowledge and skills related to community healthcare
1. I understand the role of primary care physicians.
2. I can explain home care.
3. I can explain home nursing.
4. I understand functions of primary care in community healthcare settings.
5. I can explain the comprehensive management of common and chronic diseases that are often seen in community healthcare settings.
6. I can explain the specific pathologies and illnesses unique to the elderly population.
7. I can explain interprofessional cooperation.
8. I can describe the activities of health center for disease prevention and health promotion.
9. I can explain emergency and terminal medical care provided in the community.
10. I can explain hospital-clinic or hospital-hospital collaboration.
11. I have communication skills to build good relationships through dialogue with patients and their families.
12. I have communication skills to build good relationships through dialogue with various medical, health and welfare professionals.
13. I have communication skills to build good relationships through dialogue with local people.
Items to assess students' attitudes toward community healthcare
14. Physicians engaged in community healthcare are busy.
15. I am attracted to physicians who are engaged in community health care.
16. I think practicing community healthcare is worthwhile.
17. I want to be involved in community healthcare in the future.
18. I have a desire and a sense of mission to take charge of community healthcare.
19. Where would you like to settle down and work in the future ?*

Written informed consent was obtained on the digital platform concurrently with the questionnaire after the program. The research was approved by the Ethics Committees of Kyushu University Hospital and Medical Institutions (23308-00). 

Statistical analyses

The differences in the students' perceptions before and after the program were assessed using Student's t-test. A p-value of <0.05 was considered statistically significant. All statistical analyses were performed using JMP PRO software version 17.0.0 (SAS Institute Inc., Cary, NC, USA).

## Results

Of the 216 students, 204 completed the questionnaires both before and after the program. Of those, 16 did not consent to have their responses used in this study, and 188 were included in this study (participation rate: 87.0%). The characteristics of the respondents are shown in Table 2.

**Table 2:** Demographic characteristics of the study population Age is expressed as the mean (minimum and maximum).

Characteristics	Year 2018 (n = 94)	Year 2019 (n = 94)	Total (n = 188)
Male (%)	73 (78 %)	81 (86 %)	154 (82 %)
Female (%)	21 (22 %)	13 (14 %)	34 (18 %)
Age	24.2 (23 - 32)	25.1 (23 - 40)	24.6 (23 - 40)

**Figure 2:**
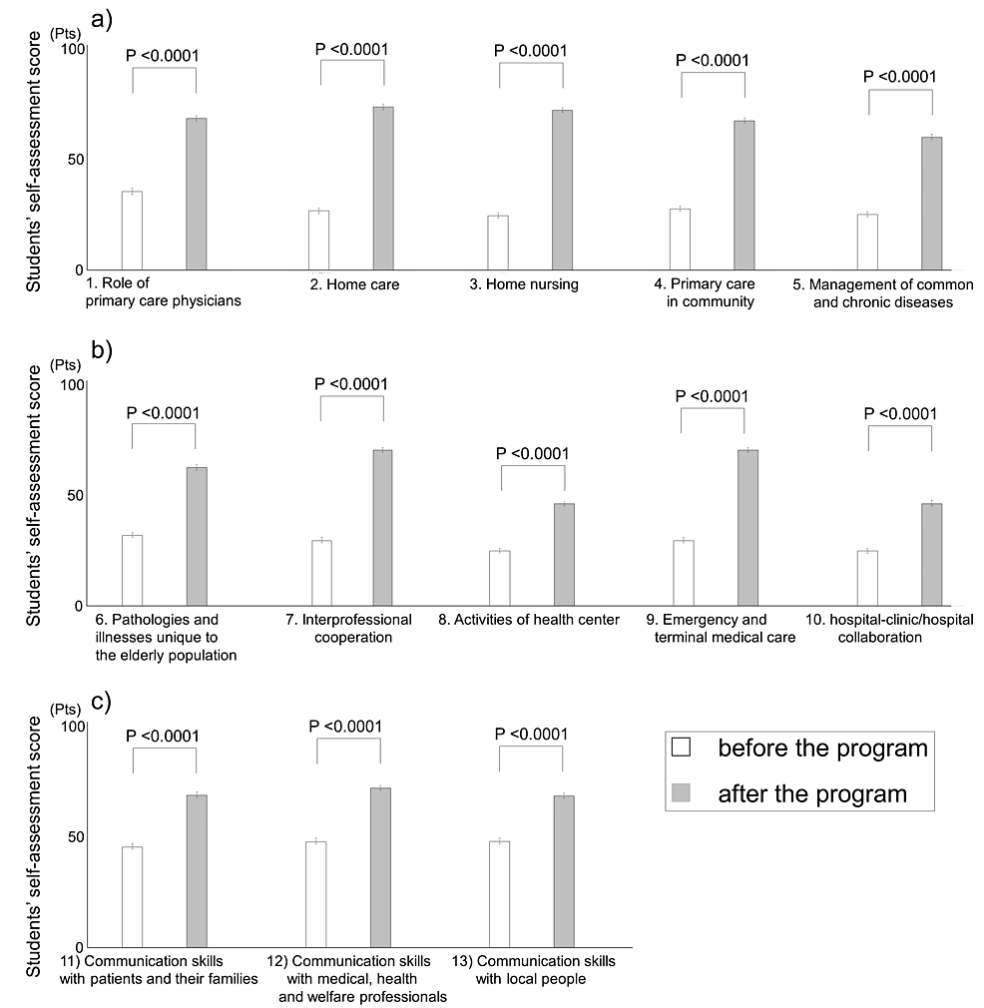
Improvements in perceptions of students' knowledge and skills by a community‐based medical education program in an urban area The mean scores of all students' self-assessments are presented in a bar chart, with error bars indicating the standard error. A p-value <0.05 was considered statistically significant using a paired t-test.

Figure 2 illustrates the pre- and post-training changes in the self-assessment of knowledge and skills relevant to community healthcare. Initially, among the 13 items, 10 scored significantly low, averaging below 40 points (Figures 2a, 2b). The assessment of communication skills across three items (Figure 2c) showed slightly higher scores than the aforementioned 10 items but still remained below 50 points. Subsequent to the training, scores for all these aspects significantly increased (P<0.0001). 

**Figure 3:**
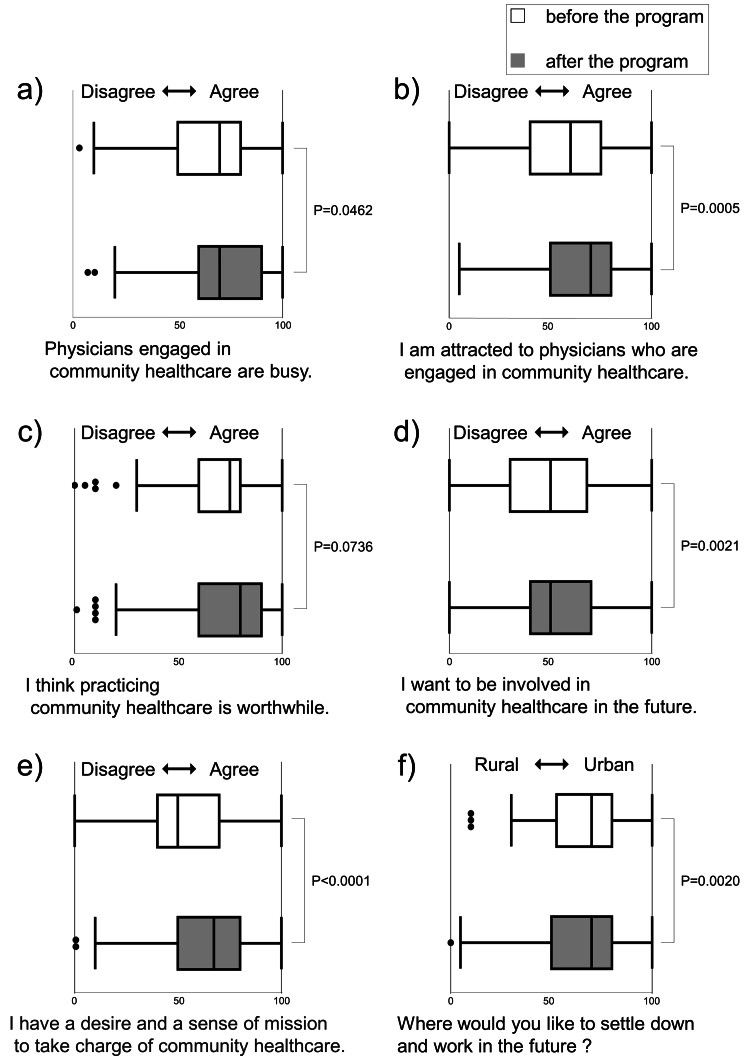
Changes in students’ attitudes toward community healthcare after a community‐based medical education program in an urban area The distribution of all students’ scores is represented by a box-and-whisker plot (median, interquartile range, and range). "0" is for "disagree" and "100" is for "agree", except in panel F, where "0" is for rural areas and "100" is for urban areas. A p-value <0.05 was considered statistically significant using a paired t-test.

Figure 3 illustrates a comparison of attitudes regarding community healthcare before and after the practical training. Initially, the attitude that 'Physicians engaged in community healthcare are busy' was notably strong (mean 68.4 points) and increased significantly after the training (Figure 3a). However, there was also a significant rise in the inclination toward attractiveness and future commitment to community healthcare as a career choice for primary care physicians (Figures 3b, 3d). Regarding satisfaction in work (Figure 3c), it initially scored high (mean 70.9 points) and experienced a slight increase after the training but did not reach statistical significance (P=0.0736). The motivation to undertake healthcare services in the region (Figure 3e) showed a particularly significant increase (P<0.0001). Figure 3f shows the students' desired work locations in the future. While the preference for urban areas remained high before and after the training (mean 68.8 points and 65.1 points, respectively), there was a significant shift towards rural areas after the training.

## Discussion

In this study, an increased understanding of medical knowledge related to community healthcare was observed even within urban community healthcare education programs. Attitudes regarding regional healthcare and primary care physicians shifted positively, demonstrating an increased inclination toward community healthcare in the future. To the best of our knowledge, existing studies on community-based medical training in urban areas are scarce. While there have been several reports from Australia detailing the process and initial issues of adapting a curriculum from a rural area to an urban setting [18,19], none of these studies thoroughly assessed the impacts on students. Our study comprehensively evaluated the effects of an urban community-based education program, covering aspects from knowledge to attitudes in a quantitative manner, distinguishing it as the first of its kind in the field.

The items listed in Figure 2, which assessed students' knowledge and skills, such as 'the role of a primary care physician,' 'home care,' 'home nursing,' and 'common and chronic diseases,' are considered important learning subjects and are also included in the Model Core Curriculum in Japan [10]. Despite most lectures and partial practical experiences completed by fifth-year students within university hospitals, the scores were remarkably low before their engagement in community-based medical training. Several previous studies have noted differences in educational content between practical experiences in university hospitals and community hospitals, suggesting the challenge of educating on community healthcare, home medical care, and patient-centered healthcare solely within university hospital settings [20,21]. It is presumed that our institution faced a similar situation. This study revealed a significant increase in scores for these topics post-training, similar to findings from previous reports in rural areas [11,20,22], highlighting the effectiveness of community healthcare education in an urban setting. Particularly noteworthy was the increased understanding of activities in public health centers, an aspect that most students had not encountered within this program. This may be attributed to students observing collaborative interactions between their practical medical facilities and public health centers. While it may suggest an advantage in experiencing comprehensive healthcare in a community setting rather than specialized care, further qualitative studies are needed to explore these reasons.

As demonstrated in Figure 3, attitudes toward community healthcare and primary care physicians working within such settings generally shifted positively after the practical training. Although Item 16 ('I think practicing community healthcare is worthwhile.') did not exhibit statistical improvement, it initially scored the highest before the program. Therefore, with a larger sample size, there might have been a potential for statistical significance. Furthermore, this study observed a significant increase in the inclination toward becoming primary care physicians in community healthcare. Primary care physicians require specific competencies [14,23]. Factors influencing medical students' career choices extend from factors determined prior to enrollment, such as age, gender, hometown, and the presence of medical relatives [7,9,24,25]. However, numerous reports indicate that exposure to rural community healthcare elevates the inclination towards community healthcare [8,11,20,24]. Similar effects could be anticipated even in urban community healthcare training programs like the one conducted in this study.

The one-week duration of our training program sets it apart from training programs at other medical schools, which are typically more extensive, lasting two weeks or longer [11,16,20], or are distributed over an extended timeframe, such as six months with four sessions per week [18,19]. While our training was short, it seemed to be as successful as previous reports. We suggested that one of the contributing factors was a balanced exposure to both clinic and hospital settings, with the choice of community hospitals offering not only acute care but also convalescent and chronic care. It enabled students to acquire a comprehensive range of knowledge within a limited timeframe. Furthermore, the decision to place students individually rather than in groups may have contributed to their positive attitude towards the training and prompted a shift in their perspective on community medicine.

In this study, there were two interesting observations regarding the attitudes of students toward community healthcare. First, despite the score increase in Item 14 ('Physicians engaged in community healthcare are busy.'), there was also an increase in the score for Item 17 ('I want to be involved in community healthcare in the future'). Previous studies have frequently highlighted 'work-life balance' as a crucial factor influencing students' career choices [24,26]. Cleland et al. reported that fifth-grade students for whom work-life balance was important were more likely to select general practice as a career choice, as revealed by a cross-sectional questionnaire survey conducted in the United Kingdom [27]. It appeared contradictory to these reports that students who completed our program tended to perceive physicians in community healthcare as busy while expressing an interest in pursuing a career in this field in the future. Further investigation is necessary to understand the relationship between career choices and how students perceive 'busyness'. Second, despite the program being in an urban setting, there was a significant increase in the inclination toward rural areas in the post-questionnaire (Item 19). These results were surprising in light of reports such as 'Observing or shadowing in an urban hospital was inversely related to family medicine residency' [28]. In this regard as well, further research is needed to investigate their sentiments towards urban versus rural settings.

This study has several limitations that should be considered when interpreting the results. First, for a program conducted within a single university, despite the participation of 26 medical facilities, it cannot be definitively claimed that the results can be extrapolated to other regions or medical schools. Second, the study relied on self-assessment data from the students, which might be subject to social desirability bias. Since the questionnaires were administered during the mandatory program, the students may have scored higher if they were concerned about the possibility of their responses being related to passing or failing. Nevertheless, precautions were taken to mitigate the influence, such as explicitly stating the right not to consent to the study on the digital platform and verbally reassuring all students during orientation that their responses would not impact their grades. Finally, this study could investigate the short-term effects of the program. Although students' preferences for primary care physicians clearly increased right after the program, it is still unclear how long the effects will continue. Further research will be needed to clarify whether the effects of community-based medical education programs increase the number of students who become primary care physicians.

## Conclusions

In this study, even within an urban community-based medical education program, there was an observed increase in attitudes toward the understanding of medical knowledge related to community healthcare. Attitudes regarding community healthcare shifted positively, and there was an increased inclination toward pursuing a career in community healthcare. The findings indicate that a community-based medical education program in an urban setting is sufficiently valuable for learning about community healthcare. This insight could assist curriculum planners in deciding on practical training locations.
